# Evaluation of diastolic function in hypertrophic cardiomyopathy and cardiac amyloidosis through semi-automatic assessment – a multi-center, multi-vendor cardiovascular magnetic resonance study

**DOI:** 10.1016/j.jocmr.2026.102705

**Published:** 2026-02-16

**Authors:** Lukas D. Weberling, Andreas Ochs, Fabian aus dem Siepen, Janek Salatzki, Ailís C. Haney, Evangelos Giannitsis, Ute Hegenbart, Stefan Schönland, Mitchel Benovoy, Benjamin Meder, Matthias G. Friedrich, Norbert Frey, Florian André

**Affiliations:** a Department of Cardiology, Angiology and Pneumology, Heidelberg University Hospital, Heidelberg, Germany; b DZHK (German Centre for Cardiovascular Research), partner site Heidelberg/Mannheim, Germany; c Department of Cardiology and Angiology, Frankfurt University Hospital, Frankfurt, Germany; d Department of Haematology, Oncology and Rheumatology, Amyloidosis Center, Heidelberg University Hospital, Heidelberg, Germany; e Area 19 Medical Inc., Montreal, Canada; f Informatics for Life, Heidelberg, Germany; g Departments of Medicine and Diagnostic Radiology, McGill University Health Centre, Montreal, Canada

**Keywords:** CMR, HCM, Amyloidosis, Atrial function, Atrial strain, Diastolic function

## Abstract

**Background and aims:**

Unlike systolic heart function, the assessment of diastolic heart function is often demanding using cardiovascular magnetic resonance (CMR) imaging. This study aims to assess the value of semi-automatic CMR-derived biplane functional parameters in patients with cardiac light chain (AL) and transthyretin (ATTR) amyloidosis or hypertrophic cardiomyopathy (HCM).

**Methods:**

This retrospective, multi-center, multi-vendor study included patients with a diagnosis of HCM or cardiac AL/ATTR amyloidosis. Proven healthy volunteers served as controls. CMRs were assessed semi-automatically to derive atrial functional parameters (volume, long axis strain, ejection fraction [EF] of both atria) and left and right atrioventricular coupling index (LACI, RACI). Subsequently, group differences after adjustment for age, sex, left ventricular (LV) EF, body mass index and presence of atrial fibrillation as well as their correlation with N-terminal pro-B-type natriuretic peptide (NT-proBNP) and New York Heart Association (NYHA) class were calculated.

**Results:**

A total of 400 participants were included (94 HCM, 95 AL, 116 ATTR, 95 controls; mean age 58 ± 15 years, 67% male (269/400)). Semi-automated analysis was feasible in all cases (median duration 34 s) with excellent reproducibility. Compared with controls, patients revealed markedly higher LACI values (40.1% vs. 15.2%, p<0.001) and impaired LA function (EF 34.3% vs. 65.2%, strain 11.5% vs. 41.5%, both p<0.001). RACI and RA function, however, distinguished disease groups: RA indices were preserved in HCM but significantly impaired in amyloidosis (RACI 41.8% vs. 20.4%; RA EF 28.8% vs. 52.5%; RA strain 12.3% vs. 34.4%, p<0.001 for all). After adjustment, both LA volume and LACI showed independent associations with NYHA class and NT-proBNP, with LACI demonstrating the strongest correlations and outperforming LVEF.

**Conclusions:**

Semi-automated biplane CMR enables fast and reproducible assessment of atrial function. Combined analysis of LACI and RACI provides clinically relevant diagnostic differentiation between HCM and cardiac amyloidosis and correlates with patient symptom burden more accurately than LVEF.

## Graphical abstract

**Figure:**
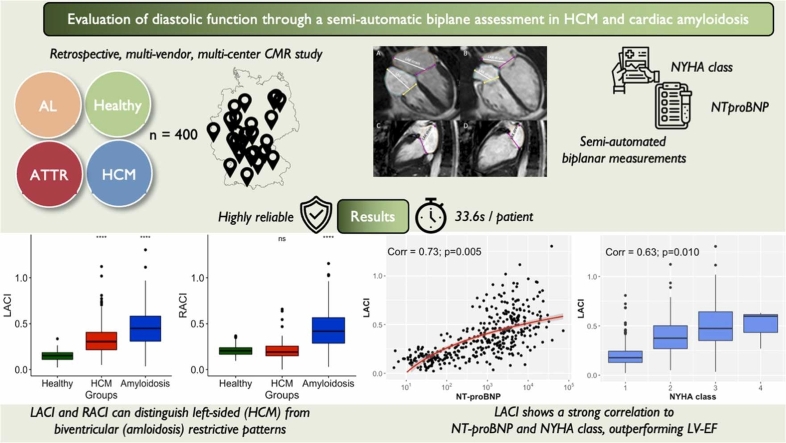

## Introduction

Cardiovascular magnetic resonance (CMR) is the current reference standard for non-invasive myocardial function characterization and endorsed by the current guidelines in the assessment of cardiomyopathies and heart failure [1], [2]. While systolic function is readily measured, the evaluation of diastolic dysfunction by CMR remains challenging. Yet, diastolic dysfunction accounts for nearly half of heart failure hospitalizations, underscoring the need for robust imaging markers [3]. Left atrial (LA) function and volume are established markers of left ventricular (LV) filling pressures and can be derived from simplified biplane CMR measurements [4], [5], [6], [7], [8], [9], [10]. Impaired LA function predicts atrial fibrillation and heart failure outcomes more accurately than LV ejection fraction [11], [12], [13], [14], [15], [16], [17]. More recently, the left atrioventricular coupling index (LACI) has been introduced to capture the interdependence of LA and LV volumes, showing prognostic relevance across populations [18], [19], [20], [21]. A right-sided equivalent (right atrioventricular coupling index, RACI) can be calculated as well, but data on that topic is scarce [22].

LA strain is another promising marker of diastolic function, being less load-dependent than volume-based indices [23], [24], [25], [26]. LA strain may be measured by feature tracking or by assessing the longitudinal shortening (%) of LA contours between the end-diastolic and end-systolic phase. The latter is often referred to as rapid strain and both methods have been shown to correlate well [10], [27], [28].

Previous studies assessing atrial function focused on outcome prediction [15], [16], [17], [20], [28], [29], [30], [31], [32], [33], [34], [35], [36], [37], [38]. Data on its diagnostic accuracy to determine diastolic function remains scarce [27], [39], [40], [41]. Previous echocardiography studies on mostly small patient groups showed a decreased LA function in hypertrophic cardiomyopathy (HCM) as well as cardiac transthyretin (ATTR) and light chain (AL) amyloidosis with the latter having the poorest function and highest LA volumes [31], [42], [43], [44], [45], [46], [47]. Importantly, right atrial (RA) function has been little studied, despite the clinical observation that RA involvement is typical of amyloidosis but uncommon in HCM.

Therefore, we aimed to evaluate the diagnostic value of a rapid, semi-automated biplane CMR approach to quantify atrial function and atrioventricular coupling indices in HCM, cardiac amyloidosis, and healthy controls, with a focus on differentiating left- from biventricular restrictive disease.

## Methods

### Study population and design

This study is a post-hoc analysis of a recently published retrospective, multi-center and multi-vendor study [48]. Four different groups (AL, ATTR, HCM, Healthy) were identified as described earlier. In short, all patient groups (AL, ATTR, HCM) consisted of patients referred to one of the specialized outpatient clinics with availability of a recent (<6 months) CMR exam and a diagnosis of either HCM or cardiac AL/ATTR amyloidosis according to international standards and guidelines [1], [49]. The study was not limited to the CMR main site but included CMR series from 55 other institutions with no limitations regarding scanner type or vendor. As controls, a group of proven healthy volunteers was drawn from a reference population, which had undergone an extensive evaluation to exclude cardiovascular diseases [50]. The study included a large variety of non-imaging biomarkers such as demographics, weight, height, symptoms, physical examination abnormalities, electrocardiography, and laboratory values. Of those, age, sex, body mass index, presence of atrial fibrillation, New York Heart Association (NYHA) class and levels of N-terminal prohormone of brain natriuretic peptide (NT-proBNP) were selected for the post-hoc analysis. In patients, disease stages were assessed using international expert consensus staging systems. For HCM, we used the system by Olivotto et al. involving various parameters like LV EF, LGE mass, symptoms or presence of rhythm disorders and the classification into nonhypertrophic, classic phenotype, adverse remodeling and overt dysfunction as stage I-IV, respectively [51]. AL patients were classified using the Mayo 2012 system, which involves the parameters NT-proBNP, troponin levels and difference in levels of light chains. Disease stage is defined as: stage I, none elevated; stage II, one elevated; stage III, two elevated; stage IV, all three elevated. Lastly, the expansion of the National Amyloidosis Centre staging system was used for ATTR involving eGFR and NT-proBNP. Here, the definition is: stage I, neither is pathological, stage II, one pathological parameter; stage III, both pathological parameter; stage IV, NT-proBNP >4000. This study was approved by the institution’s ethics committee (S-046/2022), which waived an additional informed consent.

### CMR imaging

**Fig. 1:**
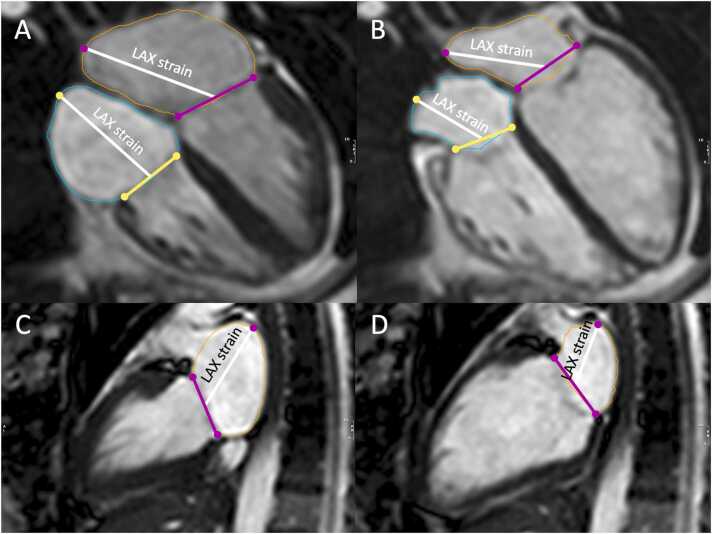
Example of the biatrial assessment to assess volumes, ejection fractions and long axis longitudinal strains (“rapid strain”, LAX) using the biplanar long axis module. Rapid strain was defined as the mean of the fractional longitudinal shortening of the atria between end-systole (A, C) and end-diastole (B, D) of the long-axis 4-chamber (A, B) and 2-chamber view (C, D).

CMR scans were carried out at our institution at a 1.5 T or 3 T MRI scanner (Achieva, IngeniaCX, Ingenia. Philips Healthcare, Best, The Netherlands) or at one of 55 different referring CMR centers on 1.5 T or 3 T scanners of the manufacturers Philips, GE HealthCare (Chicago, Illinois, United States of America) or Siemens Healthineers (Erlangen, Germany). All image analyses were performed at a core imaging lab at the University of Heidelberg (Germany) on CVI42 (version 5.14.1.2999, Circle Cardiovascular Imaging, Calgary, Alberta, Canada). Parameters were derived using the biplanar long-axis module, which performed a semi-automated analysis of 2-chamber and 4-chamber cine series. The LA appendage was excluded in the contouring. Based on the LA or RA contouring at the end-systolic and end-diastolic phase, atrial volume, atrial EF, and atrial long axis longitudinal strain (% longitudinal shortening, “rapid strain”, LAX strain) were calculated. This is illustrated in Fig. 1. LV and right ventricular (RV) end-systolic and end-diastolic volumes (EDV) along with EF were calculated using the short-axis cine stack. Contours and labels were corrected manually by one of two experienced CMR readers (L.W.; A.O.), if necessary. The LACI was calculated by the formula LACI = LAEDV / LVEDV x 100, in line with previous studies [18], [20], [21]. RACI was calculated by the formula RACI = RAEDV / RVEDV x 100. Biatrial functional parameters of 40 randomly chosen subjects (10 of each group) were analyzed a second time over 2 months after the initial assessment. Of those, 20 (5 of each group) were assessed by the same reader and 20 by a different reader to determine intra- and interrater reliability. Total analysis time including series selection, semi-automated contouring, and manual correction was noted for each of the 40 analyses.

### Statistical analysis

Statistical analysis was done using the R language and environment for statistical computing (version 4.2.1) and the R Studio user interface (R Foundation for Statistical Computing, version 2023.06.0+421, Vienna, Austria) [52]. Normal distribution was assessed using the Shapiro-Wilk test. Parametric variables are given as mean ± standard deviation and non-parametric variables as median with interquartile range. For inter- and intrarater reliability, the two-way random effects intraclass correlation coefficient (ICC 2,1) was calculated and interpreted as follows: <0.5 poor, 0.5–0.75 moderate, 0.75–0.90 good and >0.90 excellent [53]. In the initial univariable assessment of differences between groups, the Chi-squared test, Student’s test, or Mann-Whitney U test were used as appropriate and all comparisons were adjusted for age and sex. In the analysis of age- and sex-dependencies, all calculations were adjusted for disease stage. In a last step, a multivariable correlation analysis for the prediction of diastolic function was performed and involved NT-proBNP levels, NYHA class, and the biplanar CMR-derived assessment. It was adjusted for body mass index, presence of atrial fibrillation, LVEF, age and sex by fitting a generalized linear model. Correlations between NYHA class or NT-proBNP and CMR-derived parameters were tested by fitting NYHA class or NT-proBNP levels and LA/RA volume, LA/RA EF, LA/RA LAX strain, LACI, LVEF, age, sex, body mass index and presence of atrial fibrillation into one generalized linear model for each. For multiple testing, p values were adjusted using the Bonferroni correction. Correlations between parameters were assessed using the Pearson (normally distributed) or Spearman rank (not normally distributed) correlation coefficient and correlations were classified as weak (coefficient 0.1–0.29), intermediate (0.3–0.49) or strong (>0.5). The a priori significance level was set to p<0.05.

## Results

### Study population

The initial study population included 448 subjects, of whom 48 were excluded due to incomplete image data (n=39) or insufficient image quality (n=9). As a result, the final cohort comprised 400 subjects: 95 cardiac AL patients, 116 cardiac ATTR patients, 94 HCM patients, and 95 healthy volunteers. The cohort’s characteristics have been described before [48]. In summary, the patient groups unlike the control group were male dominated (72.1% (220/305) vs. 51.6% (49/95)). Both the patient and control groups encompassed a wide age spectrum, ranging from 19 to 88 years for the patient groups and from 21 to 68 years for the controls (mean age 61.5±12.8 vs. 43±12.8 years, p<0.001). Of note, the study cohort included patients of all disease stages and NYHA classes. In the HCM group, the median LGE extent was 8.9 [4.6; 15.1] %. Exertional dyspnea (78,3% (239/305)) and peripheral edema (39.0% (119/305)) were the most common symptoms and NT-proBNP was elevated in a majority of patients (83.2%). The most relevant characteristics and markers of disease stage are given in Table 1.

**Table 1:** Patient characteristics and markers of disease stage of the healthy control, HCM, AL, and ATTR groups and statistical comparisons after adjustment for age and sex. Disease stage was based on the clinical staging by Olivotto et al. for HCM, the Mayo 2012 classification for AL and the expansion of the National Amyloidosis Centre staging system for ATTR [51], [59]. Focal intramural LGE was defined as non-RV insertion point intramural Late Gadolinium Enhancement of ≤3 segments. Diffuse LGE was defined as hyperintense myocardium involving the entire circumferential extent on at least one slice.

* Since the presence of cardiovascular symptoms was an exclusion criterion for the control group (“Healthy”), markers or stages of potential disease were not compared to the patient cohorts.

*AL* cardiac light chain amyloidosis, *ATTR* cardiac transthyretin amyloidosis, *GLS* Global longitudinal strain, *LGE* Late Gadolinium Enhancement; *LV* Left ventricular; *NYHA* New York Heart Association; *RV* right ventricular, *NAC* National Amyloidosis Centre, *HCM* hypertrophic cardiom

### CMR results

**Fig. 2:**
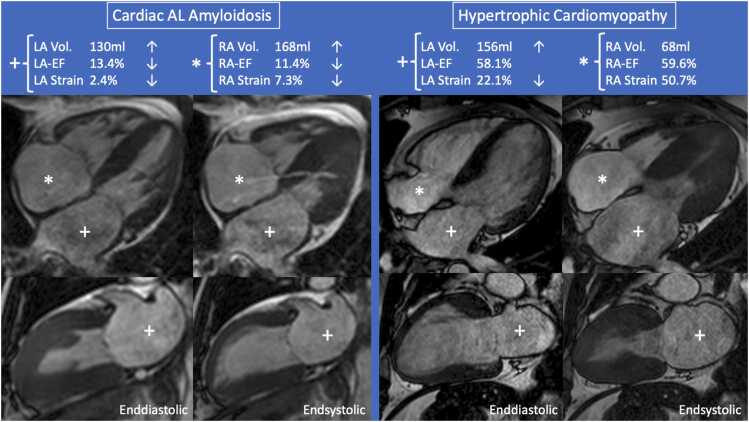
Examples of biatrial function assessment in patients with cardiac AL amyloidosis (left) and hypertrophic cardiomyopathy (right). Due to its systemic and biventricular manifestation, cardiac amyloidosis impaired both LA (+) and RA (*) functional parameters, whereas hypertrophic cardiomyopathy only reduced the LA functional parameters. *AL* cardiac light chain amyloidosis,*EF* ejection fraction, *LA* Left atrial, *LAX* long-axis, *RA* Right atrial.

Semi-automated biplane function assessment was feasible in all 400 subjects (100.0%). The median analysis time was 33.6 (24.0; 35.3) seconds in total for calculation of volume, EF, and long-axis strain of both LA and RA. Intrarater reliability was excellent for LA volume (0.98), LA EF (0.93), LA LAX strain (0.97), and RA volume (0.97) and good for RA EF (0.86), and RA LAX strain (0.89). Interrater reliability was excellent for LA volume (0.91), LA EF (0.96), LA LAX strain (0.97), and RA volume (0.98) and good for RA EF (0.85), and RA LAX strain (0.85). Fig. 2 shows an example case for both the Amyloidosis and HCM groups.

#### LACI and RACI

Healthy volunteers had the lowest LACI (15.2 [11.0; 18.5]) being significantly lower than in patients (40.1 [26.2; 55.8], p<0.001). It differed significantly between HCM (30.6 [21.5; 42.0]) and cardiac amyloidosis (44.9 [30.9; 58.3]; p<0.001), but not between amyloidosis subtypes (p=0.108). Age but not sex was a significant and independent determinant of LACI in patients (age: β=0.46 per year; p<0.001; sex p=0.798) and controls (age: β=0.12 per year; p=0.014; sex: p=0.849).

**Fig. 3:**
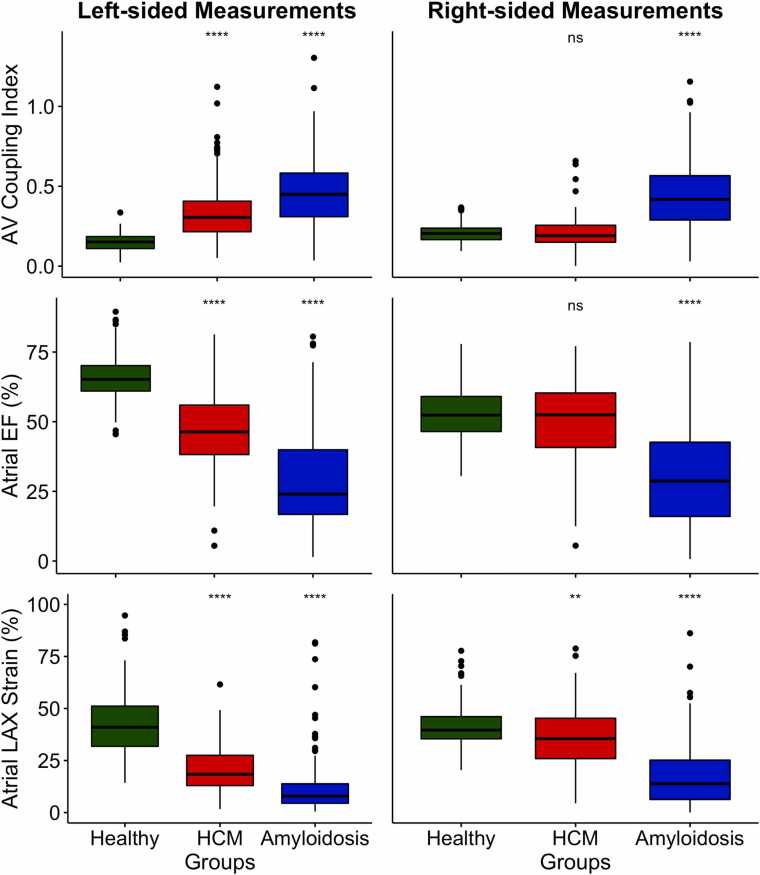
Left-sided and right-sided measurements of the atrio-ventricular (AV) coupling index, atrial ejection fraction (EF) and atrial long-axis (LAX) strain in controls (n = 95) versus patients with hypertrophic cardiomyopathy (HCM, n = 94) and amyloidosis (n = 211). Compared to healthy controls, left-sided measurements all show a highly significant (p<0.001, ****) group difference to both HCM and cardiac amyloidosis patients. In contrast, right-sided measurements are only significantly different (p<0.001, ***) in cardiac amyloidosis patients when compared to healthy controls, not in HCM patients (ns = not significant). Of note, right atrial LAX strain was slightly lower in HCM patients than in controls (p<0.01, **), but remained significantly higher than in cardiac amyloidosis patients (p<0.001).

The RACI was also lowest in controls (20.4 [16.6; 23.8]) when compared to patients. In contrast to the LACI, HCM patients (19.1 [14.9; 25.6]) showed no difference to controls (p=0.191). In contrast and similar to the LACI, cardiac amyloidosis patients had higher RACI values (41.8 [29.0; 56.9]), which were significantly higher than in both controls (p<0.001) and HCM patients (p<0.001), but with no difference between amyloidosis subtypes (p=0.942). Like the LACI, RACI was independently correlated with age in controls (β=0.14 per year; p=0.007) and patients (β=0.55 per year; p<0.001). Sex was an independent influencer of RACI in patients (β=7.9; p=0.005), but not in controls (p=0.138). LACI (p=0.202) and RACI (p=0.333) were indifferent between obstructive and non-obstructive HCM patients. Table 2, Table S1, and Fig. 3 show values of each group. Table 3 as well as Supplementary Fig. 1 summarize age and/or sex dependencies between variables.

**Table 2:** Overview of CMR-derived atrial function parameters and left/ right atrio-ventricular (AV) coupling index. Statistical differences between each group were adjusted for age and sex

* Driven only by a difference of Cardiac AL/ATTR to the controls. No statistical difference (p=0.327) was found between HCM and controls.

** Healthy controls did differ to both cardiac amyloidosis (higher RA volumes) and HCM (lower RA volumes) individually.

*AL* cardiac light chain amyloidosis, *ATTR* cardiac transthyretin amyloidosis, *AV* atrioventricular, *LA* Left atrial *LAX* long-axis, *RA* Right atrial

**Table 3:** Influence of age and sex onto CMR-derived atrial parameters and left/ right AV coupling indices. All analyses were adjusted for disease stage. Effect size for age is given per year. A positive effect size for sex indicates a higher value for male individuals. Significant p values are written in bold

*AV* atrioventricular, *LA* Left atrial, *LAX* long-axis, *RA* Right atrial

#### LA assessment

LA volume was lowest in controls (64 [56; 78] ml) with a significant difference to patients (95 [72; 121] ml, p<0.001), but no differences between patient groups (p>0.05 for all). LA EF (65.2 [61.1; 70.2] %) and LAX strain (41.5 [32.3; 54.4] %) were highest in the control group and differed significantly to all patient groups (p<0.001). Unlike LA volume, both differed significantly between HCM and cardiac amyloidosis (p<0.001) with amyloidosis showing a lower LA EF (24.0 [16.7; 39.9] %) and LA LAX strain (7.7 [4.3; 13.7] %) in comparison to HCM (46.3 [38.2; 56.0] % for LA EF, 18.4 [13.0; 27.5] % for LA LAX strain). There was no statistical difference between amyloidosis subtypes (p=0.060 for LA EF, p=0.749 for LAX strain) or between obstructive and non-obstructive HCM (p=0.088 for LA volume, p=0.169 for LA EF, p=0.399 for LA LAX strain). LA parameters are presented in Table 2 and Fig. 3, as well as Table S1.

LA volume, EF, and LAX strain were not age-dependent in healthy controls, but sex showed a significant influence onto volume (14.8 ml higher in males, p=0.001) and LAX strain (8.7% lower in males, p=0.020), but not EF. In the patient groups, all LA parameters were both sex- and age-dependent and a higher age or male sex resulted in a higher volume (0.45 ml/year, p=0.009; +19.1 ml for males, p<0.001) and a lower EF (−0.47/year, p<0.001; −4.4% for males, p=0.039) and LAX strain (−0.30/year, p<0.001; −3.7% for males, p=0.015). Detailed data are given in Table 3 as well as Supplemental Fig. 1.

#### RA assessment

Like LA volume, RA volume was highest in cardiac amyloidosis (98 [68; 126] ml) with no difference between subtypes (p=0.062) and a significant difference to healthy controls (72 [58; 87] ml). In contrast to LA volume, RA volume was significantly lower in HCM (62 [46; 79] ml) than in controls (p<0.001) and cardiac amyloidosis (p<0.001). Likewise, RA EF did not differ between healthy controls (52.4 [46.5; 59.1] %) and HCM (52.5 [40.8; 60.7] %; p=0.501) and was significantly higher than in the cardiac amyloidosis groups (28.8 [16.0; 42.8] %; p<0.001). Again, no difference was found between amyloidosis subtypes (p=0.423). For RA LAX strain, highest values were again found in the HCM (39.2 [35.1; 45.6] %) and control group (34.4 [24.7; 44.8] %), both statistically higher than the cardiac amyloidosis groups, which exhibited very low RA LAX strain values (12.3 [5.4; 23.9] %; p<0.001 for both). There was no difference between HCM and controls (p=0.148), but a significant difference between amyloidosis subtypes (AL: 17.0 [7.1; 29.4] %; ATTR: 9.1 [4.1; 19.7] %; p=0.043). Detailed data are given in Table 2 and Fig. 3. RA variables did not differ between obstructive and non-obstructive HCM patients (p=0.289 for RA volume, p=0.585 for RA EF, p=0.545 for RA LAX strain, Table S1).

RA EF was neither age- nor sex-dependent in healthy controls, whereas RA volume was sex- (+24.9 ml for males, p<0.001) and RA LAX strain was age-dependent (−0.21%/year, p=0.013). In the patient groups, all RA parameters were both age- and sex-dependent. Similar to the LA assessment, advanced age and male sex was associated with higher volumes (0.88 ml/year, p<0.001; +33.3 ml for males, p<0.001) and lower EF (−0.35/year, p<0.001; −8.2% for males, p=0.001) and LAX strain (−0.37/year, p<0.001; −8.0% for males, p<0.001). Detailed data are presented in Table 3 and Supplementary Fig. 1.

### Predictors of diastolic function

The first generalized linear model contained (I) adjusting factors, (II) NYHA class and (III) volume, coupling index, LAX strain and ejection fraction of both atria. After adjustment for LV EF, age, sex, body mass index and presence of atrial fibrillation, the factors LA volume (p<0.001), LACI (p=0.010), and LAEF (p<0.001) remained independently correlated with NYHA class of participants. Of the adjustment factors, only age was significantly correlated to NYHA class (p<0.001). The correlation was strong for LACI (correlation coefficient 0.63), intermediate for LA volume (0.32), and strong for LA EF (−0.70). LACI and LA EF outperformed LV EF (−0.36) in their correlation to NYHA class. No significant correlation was found for RA volume, RA EF and both RA/LA LAX strain.

**Fig. 4:**
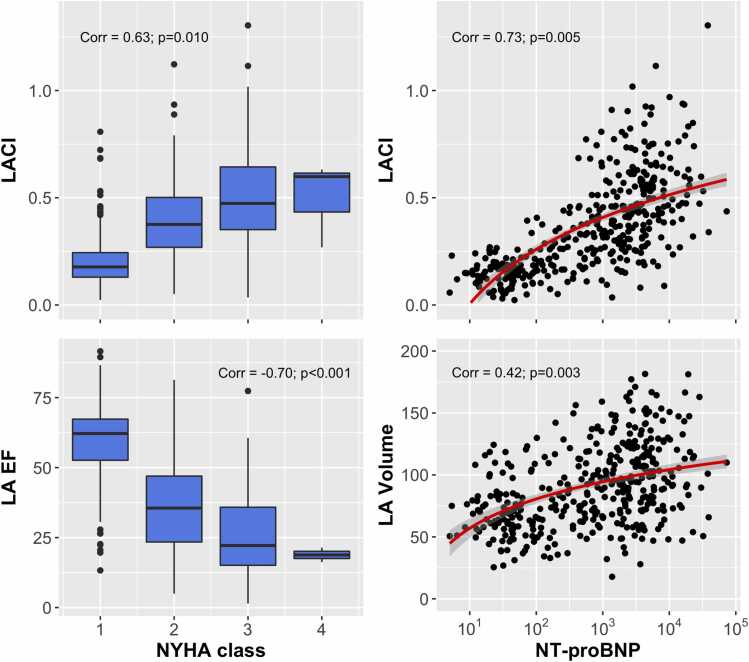
Left: Correlation of the left atrioventricular coupling index (LACI) and the left atrial ejection fraction (LA EF) with the New York Heart Association (NYHA) class. Right: Correlation of the left atrioventricular coupling index (LACI) and the left atrial volume (LA Volume) with NT-proBNP levels. *NT-proBNP* N-terminal pro-B-type natriuretic peptide

The second generalized linear model contained (I) adjusting factors, (II) NT-proBNP levels and (III) volume, coupling index, LAX strain and ejection fraction of both atria. Here, LA volume and LACI were also significantly and independently correlated with blood NT-proBNP levels of participants (p=0.003; p=0.005) after adjustment for LV EF, age, sex, body mass index and presence of atrial fibrillation. Of the adjustment factors, only LV EF was also significantly and independently correlated with NT-proBNP levels (p<0.001). No independent correlation was found for RA parameters, LA EF and LA LAX strain. The correlation was intermediate for LA volume (correlation coefficient 0.42) and strong for LACI (0.73), both superior to LV EF (0.41). An illustration of all correlations is given in Fig. 4.

In a subgroup analysis of the HCM group, extent of LGE of total mass (%) correlated with LAEF (effect size −0.11%; p=0.042) and LA LAX strain (effect size −0.15%; p=0.047) individually, but parameters did not reach levels of significance in a multivariable analysis.

To determine whether the initial findings were predominantly influenced by advanced disease stages, we conducted a sensitivity analysis limited to patients with early-stage disease (stages I/II), thereby assessing the robustness of the conclusions and model performance in this subgroup. The univariable analysis revealed an identical performance and can be found in Table S2. The only difference was a marked difference of values between the early-stage AL and early-stage ATTR group with all parameters reaching a statistical difference which was not the case for the whole cohort. This may be attributed to different stage definitions of early disease in AL and ATTR, e.g. an NTproBNP of 1900ng/ml and eGFR of 44 ml/min without any other risk factor would be considered a stadium I in AL but a stadium III in ATTR. In the first generalized linear model LA volume (p<0.001), LACI (p=0.008) and LAEF (p=0.016) remained significant predictors of NYHA class in this subgroup, whilst the second generalized model revealed a continuing correlation of LAEF (p=0.013) with NTproBNP levels. Of the adjusting factors, age remained significantly associated with NYHA class (p=0.006) and NTproBNP levels (p=0.017), whilst atrial fibrillation was only associated with NTproBNP levels (p=0.005).

## Discussion

This multi-center, multi-vendor study of 305 cardiomyopathy patients and 95 controls demonstrated that semi-automated CMR biatrial analysis can capture diastolic dysfunction and differentiate left- from biventricular restrictive patterns. Several key findings have direct clinical relevance.

First, semi-automated CMR assessment of biatrial function and LACI/RACI was fast with a median analysis time of approximately 30 s and featured a high intra- und interrater reliability/reproducibility. This underscores its potential for integration into routine clinical workflows.

Second, we identified a distinct diagnostic profile: while left atrial dysfunction was present in both HCM and cardiac amyloidosis, right atrial impairment was specific to amyloidosis. Of note, this also held true in a sensitivity analysis of early-disease (stage I/II) patients. HCM patients did not have abnormal right atrial function or volumes and, thus, were not significantly different from healthy controls in this regard. Importantly, this is the first and largest CMR study to establish this pattern, complementing smaller echocardiographic observations [40], [41]. Similarly, LACI was elevated in both cardiac amyloidosis and HCM, whereas RACI was only elevated in cardiac amyloidosis. Thus, it may help to better understand the heart failure phenotype beyond systolic function alone and to differentiate between HCM and cardiac amyloidosis, between which misdiagnoses are common [42], [54], [55]. This study is one of the first to introduce the RACI as a right-sided equivalent of the LACI. The differences found here between HCM and cardiac amyloidosis highlight the potential usefulness of the RACI as a rapid method of assessing right-sided restriction [22]. However, between different causes of right-sided restriction, especially between different subtypes of cardiac amyloidosis, the analysis did not show sufficient discriminatory power, as only RA LAX strain showed a borderline significant difference between groups. (p=0.043). In a small echocardiographic study by Versteylen et al. comparing cardiac amyloidosis subtypes, the observation that atrial volumes and ejection fraction are indifferent between subtypes was similar to our study. In contrast to our study, the RA LAX strain was indifferent between subtypes whilst they report a borderline significant difference between the LA strain of both groups. However, NTpro-BNP and Troponin I levels differed largely between the two groups in their study raising the question if their patient cohort is comparable to ours [43]. Also, their analysis was not adjusted for confounders and used another modality, both potentially confounding results [43]. The smaller case numbers might explain the indifference of the RA LAX strain. A more exact amyloidosis subgroup distinction however necessitates the inclusion of more CMR biomarkers [48].

Third, the rapid assessment showed to be a promising tool to assess diastolic function with CMR and therefore may lead to a better understanding of the patient’s symptom burden beyond systolic LV function alone. Both LACI and LA volumes correlated significantly with both NYHA class and NT-proBNP levels after adjustment for LV EF, age, sex, body mass index and presence of atrial fibrillation. For both, LACI showed a strong correlation superior to that of LV EF. This is in accordance with previous smaller studies on patients with HCM, heart failure with preserved ejection fraction, atrial fibrillation and coronary artery disease [23], [27], [45]. In contrast to some of the previous imaging studies of atrial strain, our study focused on the global LA LAX strain rather than its components (booster, reservoir, conduit strain) because it is easier to calculate and therefore more practical to use [31]. To that regard, the excellent inter- and intrarater reliability found in our study also promotes usage of the global LA LAX strain. Echocardiography will remain the primary modality for assessing left-sided restriction due to its ease and speed of access and profound validation. However, the role of echocardiography for right-sided restriction is less established and the better visualization of the RA and RV by CMR is an advantage. Also, a relevant degree of variability of echocardiography measurements represents a disadvantage when compared to CMR [56].

## Limitations

There are some limitations to consider. One is the retrospective design chosen due to the relatively low prevalence of the assessed cardiomyopathies. Also, the findings in this study might not be applicable to certain patient groups, such as implantable cardiac device carriers or non-white ethnicities, which were both underrepresented in this study. Specifically in the HCM group, patients with outflow tract obstruction were also underrepresented, which might be explained by the fact that these patients are more easily diagnosed by echocardiography and seldom referred to CMR for further evaluation. The multi-center design with data collection from 56 institutions resulted in the exclusion of 39 of a total of 448 patients (8.7%) since they did not include the required minimum CMR data set of three short-axis slices and three long-axis cine views (4-/3-/2-chamber). This underlines the need for standardized CMR protocols endorsed by the current guidelines and the Society of Cardiovascular Magnetic Resonance [57]. In contrast, only 2% (9/448) of examinations were excluded for insufficient image quality, despite a relevant prevalence of atrial fibrillation or overweight. To that regard, parametric mapping data was predominantly not available and would have been of interest as it plays a key role in amyloidosis assessment. Furthermore, a comparison of measurements with invasively derived hemodynamic parameters and a full functional capacity assessment including spiroergometry would have been desirable but was not available. A previous echocardiography study in 322 patients showed LA strain to correlate well with invasively measured filling pressures [5]. Another study used CMR and an artificial intelligence model to successfully predict filling pressures [58]. However, the biatrial assessment included only atrial diameter, not function or volume. Another limitation of our study is the missing comparison to echocardiography measurements, which was not feasible due to the multi-center design of the study with no standardization regarding image acquisition or assessment at most participating outpatients’ cardiologists. Furthermore, a variation of atrial measurements with volume status is to be expected and aligns well with our found correlation to NT-proBNP and NYHA class. A previous echocardiography study on healthy controls by Genovese et al. found left-atrial strain however to be less load-dependent than atrial volume [26]. The load dependence of atrial measurements in patients with heart failure remains to be determined and is expected to be lower than in controls. Yet, all of our patients were non-hospitalized and presented at an elective CMR appointment. Furthermore, the reported variations of atrial measurements in response to preload variation previously reported were much lower than the group differences in our study. Lastly, for the group differences of atrial measurements between HCM, AL and ATTR potentially varying disease stages need to be considered. Adjusted for age and sex, NYHA class and disease stage differed between the three (Table 1). However, a sensitivity analysis in exclusively early stage (I/II) patients did not change the results. Also, heart failure encompasses a broad spectrum of contributors from systolic function, diastolic function, outflow tract obstruction to chronotropic incompetence of which each entity has its one individual profile. The here-assessed atrial measurements have a promising potential in the assessment of previously overlooked diastolic function and can help to distinguish left-sided from right-sided restriction. However, a precise distinction of these entities is reliant on the inclusion of ventricular biomarkers, as we have previously demonstrated in this cohort [48].

## Conclusion

In conclusion, semi-automated, biatrial CMR provides a fast and reliable tool for complementing the evaluation of cardiomyopathies. The RACI as right-sided equivalent of the LACI aids in the differentiation of HCM from cardiac amyloidosis. These findings may help to assess diastolic function as well as right- and left-sided restriction using CMR. Derived parameters show an independent and stronger correlation with NYHA class and NT-proBNP levels than LVEF.

## Funding

The initial study was a collaboration project between the Heidelberg University Hospital, Pfizer and Circle Cardiovascular Imaging, as detailed in the study manuscript [48]. This post-hoc analysis however was not part of this collaboration project and did not receive any funding.

## CRediT authorship contribution statement

**Fabian aus dem Siepen:** Writing – review and editing, Resources, Data curation, Conceptualization. **Benjamin Meder:** Writing – review and editing, Resources, Data curation. **Friedrich Matthias Gero:** Writing – review and editing, Supervision, Resources, Project administration, Funding acquisition, Conceptualization. **Norbert Frey:** Writing – review and editing, Supervision, Resources, Funding acquisition. **Weberling Lukas Damian:** Writing – review and editing, Writing – original draft, Visualization, Validation, Resources, Project administration, Methodology, Investigation, Formal analysis, Data curation, Conceptualization. **Florian André:** Writing – original draft, Visualization, Validation, Supervision, Project administration, Funding acquisition, Conceptualization. **Andreas Ochs:** Writing – review and editing, Investigation, Formal analysis, Data curation. **Evangelos Giannitsis:** Writing – review and editing, Resources, Funding acquisition, Data curation. **Ute Hegenbart:** Writing – review and editing, Resources, Data curation. **Stefan Schönland:** Writing – review and editing, Resources, Data curation. **Mitchel Benovoy:** Writing – review and editing, Software, Methodology, Conceptualization. **Janek Salatzki:** Writing – review and editing, Investigation, Formal analysis. **Haney Ailís Ceara:** Writing – review and editing, Investigation, Formal analysis.

## Declaration of Competing Interest

The authors declare the following financial interests/personal relationships which may be considered as potential competing interests: F.S., U.H., S.S., B.M., N.F. reports a relationship with Pfizer Inc that includes: consulting or advisory, speaking and lecture fees, and travel reimbursement. F.S., U.H., S.S. reports a relationship with Janssen Pharmaceuticals Inc that includes: consulting or advisory, funding grants, speaking and lecture fees, and travel reimbursement. F.S. reports a relationship with Ionis Pharmaceuticals Inc that includes: speaking and lecture fees. F.S., U.H. reports a relationship with Alnylam Pharmaceuticals Inc that includes: consulting or advisory and speaking and lecture fees. E.G. reports a relationship with Roche Diagnostics Corporation that includes: consulting or advisory, funding grants, and speaking and lecture fees. E.G., U.H., S.S., B.M., S.S. reports a relationship with AstraZeneca Pharmaceuticals LP that includes: speaking and lecture fees and travel reimbursement. E.G., N.F. reports a relationship with Bayer Corporation that includes: funding grants and speaking and lecture fees. E.G., B.M. reports a relationship with Daiichi Sankyo Inc that includes: funding grants, speaking and lecture fees, and travel reimbursement. E.G. reports a relationship with Eli Lilly and Company that includes: speaking and lecture fees. E.G. reports a relationship with BRAHMS AG that includes: consulting or advisory and funding grants. E.G. reports a relationship with Thermo Fisher Scientific Inc that includes: consulting or advisory and funding grants. E.G., N.F. reports a relationship with Boehringer Ingelheim GmbH that includes: consulting or advisory and speaking and lecture fees. M.B., M.F. reports a relationship with Area 19 Medical that includes: board membership and equity or stocks. U.H. and S.S. reports a relationship with Prothena Corporation plc that includes: consulting or advisory, funding grants, speaking and lecture fees, and travel reimbursement. UH and SS reports a relationship with Alexion that includes: consulting or advisory, funding grants, and speaking and lecture fees. U.H. and S.S. reports a relationship with Neurimmune AG that includes: consulting or advisory and funding grants. S.S. reports a relationship with Sanofi that includes: funding grants. S.S. reports a relationship with Protego that includes: funding grants. S.S. reports a relationship with Telix Pharmaceuticals Limited that includes: consulting or advisory. S.S. reports a relationship with Sobi Inc that includes: speaking and lecture fees. S.S. reports a relationship with Takeda Pharmaceutical Company Limited that includes: speaking and lecture fees. S.S. reports a relationship with Celgene Corporation that includes: travel reimbursement. S.S. reports a relationship with The Binding Site Group Limited that includes: travel reimbursement. S.S. reports a relationship with Jazz Pharmaceuticals Inc that includes: travel reimbursement. B.M. reports a relationship with Bristol Myers Squibb Co that includes: consulting or advisory, speaking and lecture fees, and travel reimbursement. B.M. reports a relationship with MyoKardia Inc that includes: speaking and lecture fees and travel reimbursement. B.M. reports a relationship with Novartis that includes: speaking and lecture fees and travel reimbursement. B.M. reports a relationship with Apple Inc that includes: speaking and lecture fees and travel reimbursement. B.M. reports a relationship with Novo Nordisk Inc that includes:. M.G.F. reports a relationship with Circle Cardiovascular Imaging Inc that includes: equity or stocks. The initial study was a collaboration project between the Heidelberg University Hospital, Pfizer and Circle Cardiovascular Imaging, as detailed in the already published study manuscript. This post-hoc analysis however was not part of this collaboration project and did not receive any funding. If there are other authors, they declare that they have no known competing financial interests or personal relationships that could have appeared to influence the work reported in this paper.

## Data Availability

Upon request, and subject to review, the corresponding author will provide data that support the findings of this study. Subject to certain criteria, conditions and exceptions, the corresponding author may also provide access to the related individual de-identified participant data. Contact the corresponding author for more information.

## Appendix A Supplemental material

Supplemental material Supplemental Table 1: Subgroup characteristics of the HCM group between those with LV outflow tract obstruction (HOCM) und those without (HNCM). Data is presented as median [interquartile range] or as frequencies, as appropriate. AV = atrioventricular; GLS = Global longitudinal strain; LA = Left atrial; LAX = long-axis; LV = Left ventricular; NYHA = New York Heart Association; RA = Right atrial. Supplemental Table 2: Performance of the left and right atrio-ventricular (AV) indices as well as the atrial assessment in a subgroup of patients at an early stage of their disease (stage I and II). Disease stage was based on the clinical staging by Olivotto et al. for HCM, the Mayo 2012 classification for AL and the expansion of the National Amyloidosis Centre staging system for ATTR [51], [59]. AL = cardiac light chain amyloidosis; ATTR = cardiac transthyretin amyloidosis; AV = atrioventricular; LA = Left atrial; LAX = long-axis; RA = Right atrial

.

Supplemental material Supplemental Fig. 1: Portrayal of age and group dependency for the atrioventricular coupling index (upper row), the atrial ejection fraction (middle row) and the atrial long axis strain (lower row) of the left (left column) and right (right column) atria according to patient group or healthy controls (n = 95). AL = light chain amyloidosis (n = 95); ATTR = transthyretin amyloidosis (n = 116); EF = ejection fraction; HCM = hypertrophic cardiomyopathy (n = 94); LA = left atrial; RA = right atrial
