# Supplementary figures and images for: Evaluation of diastolic function in hypertrophic cardiomyopathy and cardiac amyloidosis through semi-automatic assessment – a multi-center, multi-vendor cardiovascular magnetic resonance study
Source: J Cardiovasc Magn Reson. 2026 Feb 16;28(1):102705. doi: 10.1016/j.jocmr.2026.102705 (PMC13208777; doi:10.1016/j.jocmr.2026.102705)

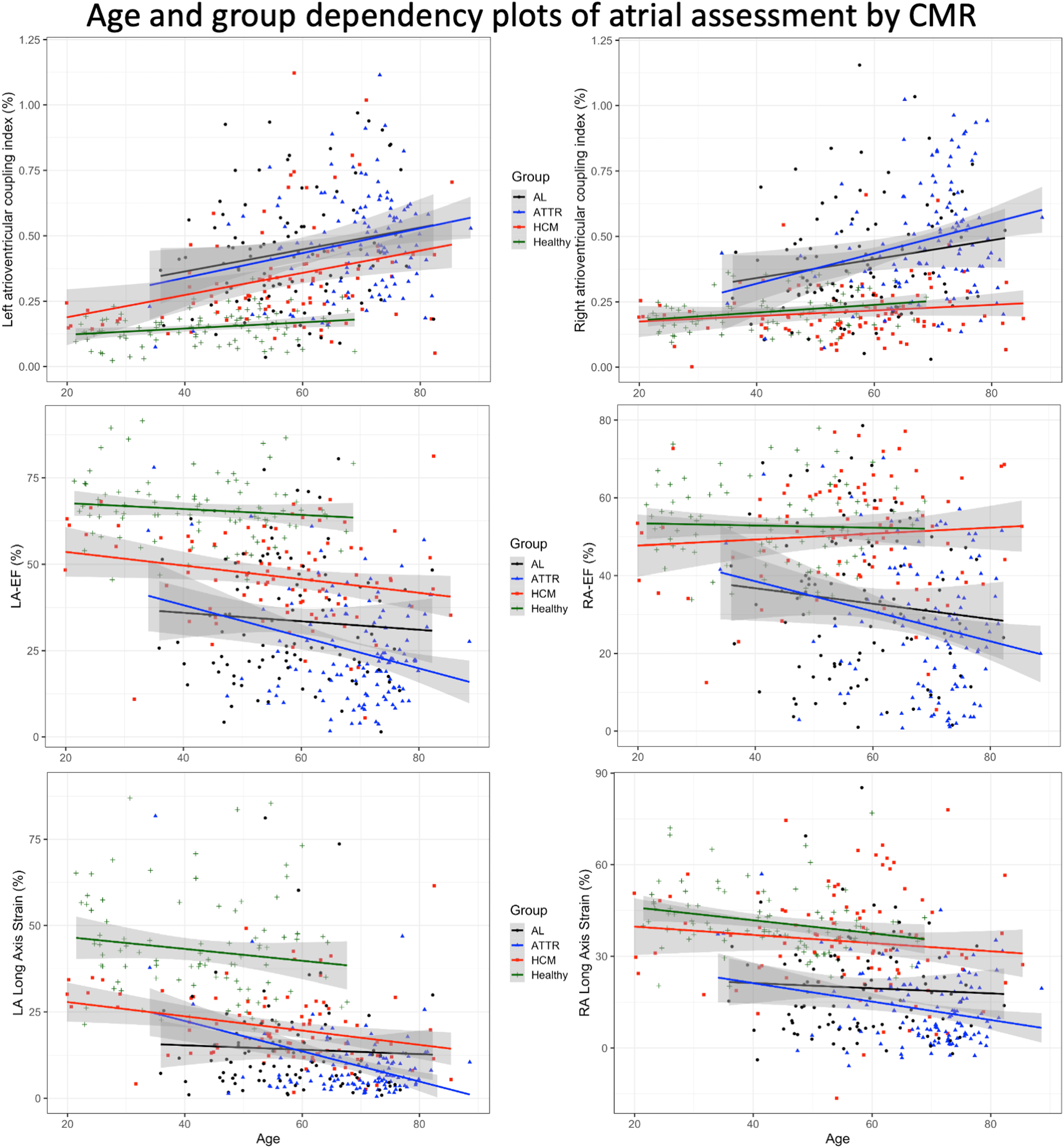

Supplement: Supplementary file 2 — Supplemental material Supplemental Fig. 1: Portrayal of age and group dependency for the atrioventricular coupling index (upper row), the atrial ejection fraction (middle row) and the atrial long axis strain (lower row) of the left (left column) and right (right column) atria according to patient group or healthy controls (n = 95). AL = light chain amyloidosis (n = 95); ATTR = transthyretin amyloidosis (n = 116); EF = ejection fraction; HCM = hypertrophic cardiomyopathy (n = 94); LA = left atrial; RA = right atrial [file mmc2.jpg]
